# Ewald sphere correction using a single side-band image processing algorithm

**DOI:** 10.1016/j.ultramic.2017.11.001

**Published:** 2018-04

**Authors:** Christopher J. Russo, Richard Henderson

**Affiliations:** MRC Laboratory of Molecular Biology, Francis Crick Avenue, Cambridge CB2 0QH, UK

**Keywords:** Ewald sphere, Single-particle reconstruction, CTF, Depth of field, cryoEM, Structure determination

## Abstract

Curvature of the Ewald sphere limits the resolution at which Fourier components in an image can be approximated as corresponding to a projection of the object. Since the radius of the Ewald sphere is inversely proportional to the wavelength of the imaging electrons, this normally imposes a limit on the thickness of specimen for which images can be easily interpreted to a particular resolution. Here we present a computational method for precisely correcting for the curvature of the Ewald sphere using defocused images that delocalise the high-resolution Fourier components from the primary image. By correcting for each approximately Friedel-symmetry-related sideband separately using two distinct complex transforms that effectively move the displaced Fourier components back to where they belong in the structure, we can determine the amplitude and phase of each of the Fourier components separately. This precisely accounts for the effect of Ewald sphere curvature over a bandwidth defined by the defocus and the size of the particle being imaged. We demonstrate this processing algorithm using: 1. simulated images of a particle with only a single, high-resolution Fourier component, and 2. experimental images of gold nanoparticles embedded in ice. Processing micrographs with this algorithm will allow higher resolution imaging of thicker specimens at lower energies without any image degradation or blurring due to errors made by the assumption of a flat Ewald sphere. Although the procedure will work best on images recorded with higher defocus settings than used normally, it should still improve 3D single-particle structure determination using images recorded at any defocus and any electron energy. Finally, since the Ewald sphere curvature is in a known direction in the third dimension which is parallel to the direction of view, this algorithm automatically determines the absolute hand of the specimen without the need for pairs of images with a known tilt angle difference.

## Highlights

• A method for correcting for the curvature of the Ewald sphere is presented.
• We demonstrate the algorithm using gold nanoparticles embedded in ice.
• The algorithm automatically determines the absolute hand of the specimen.
• It allows higher resolution imaging of thicker specimens at lower energies.
• It should also improve all high resolution structures determined by cryoEM.

## 1 Introduction

**Fig. 1:**
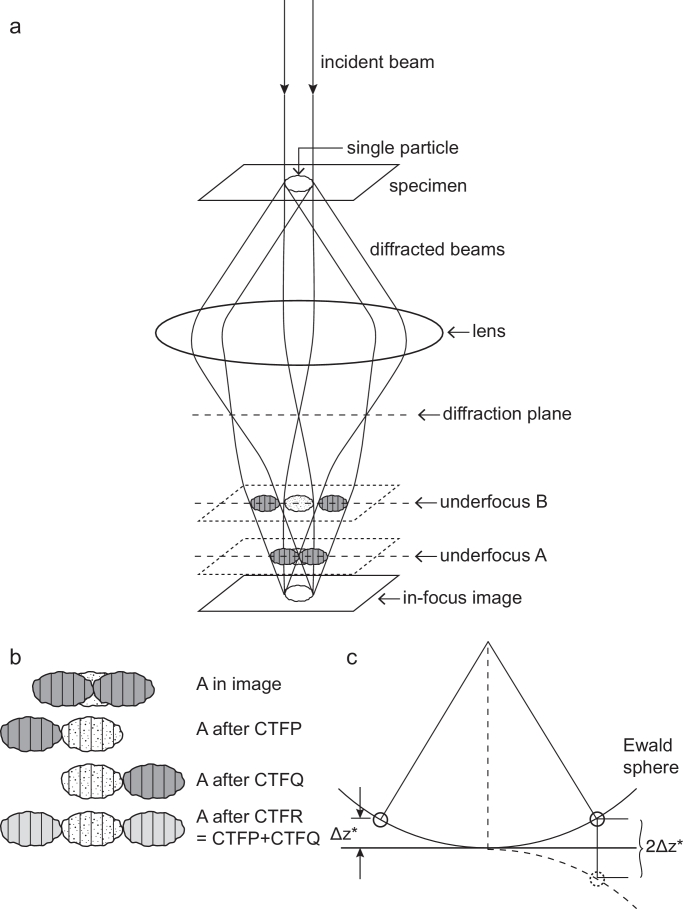
(a) Diagram showing how underfocused images keep two approximately Friedel-related diffraction patterns completely separate so that they do not overlap in the image. Since they do not overlap physically in the image, their amplitude and phase can be recovered by an appropriate image processing procedure. At underfocus B, the two diffracted beams are fully resolved from the low-resolution shadow of the particle, whereas underfocus A is the minimum underfocus needed to avoid any overlap of the high-resolution information. (b) The result of applying the two *CTFP* and *CTFQ* procedures is shown in comparison with the result of applying conventional *CTF* correction. The conventional *CTF* correction, termed *CTFR*, is equivalent to the application of CTFP+CTFQ. (c) Diagram of the Ewald sphere construction, and how the deviation from the central section, *Δz*^⋆^, is calculated. The two diffracted beams are shown intersecting the Ewald sphere on the right and left sides of the diagram. The intersection to the left is also shown as its Friedel-related partner, dashed, on the right. The 2*Δz*^⋆^ distance is the separation of the two Fourier components in reciprocal space.

One of the outstanding problems in high resolution electron cryomicroscopy (cryoEM) arises from the fact that near-focus images superimpose the fringes from Fourier components created by interference between the unscattered beam and the two beams scattered from the specimen in opposing directions from the beam axis (Fig. 1(a)). These two approximately Friedel-related Fourier components differ slightly in both amplitude and phase because they arise from Bragg reflection in slightly different directions, so they sample different positions in the 3D Fourier transform of the object being examined. Once recorded as an in-focus image at the detector, it is not possible to know which diffracted beam created the resulting Fourier component, nor in what proportion, since they are indistinguishable and overlap precisely in real and reciprocal space. The geometry of this diffraction was elegantly described by Ewald, in the context of X-ray diffraction, using a construction (the Ewald sphere) where the unscattered and elastically scattered beams are drawn as radii of a sphere in reciprocal space with radius equal to the reciprocal of wavelength (Fig. 1(c)). At larger values of defocus, however, the two Fourier fringes become physically separated in the image (Fig. 1(a)), thus suggesting a procedure (Fig. 1(b)) for the separate determination of the amplitudes and phases of the two respective Fourier components. In effect, each Fourier component is shifted radially from the centre of the particle from which it arose by an amount dependent on the electron-optical properties of the objective lens, essentially the amount of defocus and spherical aberration, although other factors such as beam tilt are also involved. Extraction of the amplitudes and phases of the Fourier components from a highly defocussed image can in principle be done in many ways. One possibility would be to extract the information from the appropriate local area of the raw image and apply a correction for the phase shift caused by its displacement from its origin at the centre of the particle. This would be a computationally inefficient procedure since it would require the extraction of a boxed area from the image plus a Fourier transformation that would have to be carried out separately for each of many thousands of Fourier components. Instead, we would like to have a simple procedure that treats the whole image with a small number of computations that allow extraction of all the amplitudes and phases in parallel.

In conventional contrast transfer function (*CTF*) correction, the procedure of multiplication of the Fourier transform of the whole image by a two-dimensional function that describes the *CTF* provides this simple and efficient computation [1], [2], but is valid only for a flat Ewald sphere. In this paper, we describe a similar single side-band correction procedure that is computationally only slightly less efficient but allows the separate determination of the amplitudes and phases of the two pseudo-Friedel-related Fourier components, provided the images are recorded with a sufficiently high value for the defocus. In an earlier paper by Wolf et al. [3], the use of a single side-band correction was also proposed and tested on simulated images. The procedure described here involves a similar single side-band algorithm, but additionally includes a subsequent masking step that removes any contribution from the incorrect, pseudo-Friedel-related Fourier component, advocates the use of sufficient defocus to avoid overlap of the these components in any desired bandwidth, and describes the relationship (and thus imaging parameters required for optimal results) between particle diameter, electron wavelength, defocus and box size.

## 2 Materials and methods

### 2.1 Single-sideband CTF correction: Theory

In bright-field defocused phase-contrast images, the approximately Friedel-related Fourier components scattered from a specimen particle are physically separated along trajectories that follow their respective Bragg scattering angles and are rendered separate on the micrograph by an amount that increases proportionally with resolution (Fig. 1(a)). The two components appear on opposite sides of the particle, with their centres separated by a distance 2*ΔFλ*/*d*, where *ΔF* is defocus, *λ* is the electron wavelength, and *d* is resolution in units of distance (see Fig. 1). To ensure that the two approximately Friedel-related Fourier components from the particle do not overlap, their separation must be greater than the particle diameter *D*. This corresponds to a defocus greater than *Dd*/2*λ*. For 300 keV electrons with λ=0.02 Å, the Fourier components beyond 4 Å resolution are separated provided the defocus is greater than 1 µm for a 100 Å particle, or greater than 10 µm for a 1000 Å diameter particle. As a result, the two Fourier components, which sample reciprocal space in positions that deviate slightly from the central section that corresponds to a projection in the direction of the unscattered beam, due to Ewald sphere curvature, can be treated separately. This then allows a simple procedure to account for Ewald sphere curvature.

Computationally, the procedure can be implemented by the separate application of two complex-valued, single-sideband corrections – one for each of the two approximately Friedel-related Fourier components. In normal *CTF* correction, these single side-band corrections are summed to give a contrast transfer function that is real. We refer to the conventional, real-valued contrast transfer function as *CTFR* and the two opposing, single-sideband corrections as *CTFP* and *CTFQ*. We begin by writing an expression for the scattering-angle dependent phase shift of the electron wave caused by the objective lens

(1) $$\chi =-\frac{2\pi }{\lambda }\left(\Delta F\frac{\theta^{2}}{2}-C_{s}\frac{\theta^{4}}{4}\right)$$

where *ΔF* is the defocus, *C_s_* is the third order spherical aberration coefficient of the lens, and *θ* is the diffraction angle ( ≃ *λ*/*d*). We then define two single-sideband contrast transfer functions as

(2) $$CTFP=e^{+i(\chi +\pi /2)}$$

(3) $$CTFQ=e^{-i(\chi +\pi /2)}$$

where the addition of *π*/2 is the phase shift upon scattering by the specimen. Note that additional terms like two-fold astigmatism, axial coma, etc. could also be included in (1) to account for other aberrations in the lens. Note also that these two complex functions, when summed, are equal to the conventional contrast transfer function, *CTFR*. To prove this equivalence, we take

CTFR=CTFP+CTFQ

and substitute using (2) and (3) to get

(4) $$\begin{array}{c} \begin{array}{cc} CTFR & =e^{+i(\chi +\pi /2)}+e^{-i(\chi +\pi /2)}\\ & =2\cos(\chi +\pi /2)\\ & =-2\sin\chi \end{array} \end{array}$$

which is the conventional expression for the contrast transfer function. We have not included amplitude contrast explicitly in the above Eqs. (2)–(4) for clarity. In practice this should be included either by adding a correction to the π/2=90° phase shift in Eqs. (2) and (3) to be  ≃  94° (for 100 keV), or by adding an amplitude term to replace −sinχ by $W\cos\chi -\sqrt{1-W^{2}}\sin\chi ,$ where *W* ≃ 0.07 for 100 keV electrons [4] and scales to *W* ≃ 0.04 for 300 keV electrons.

To perform the single-sideband correction for Fourier components on one side of the particles in the micrograph, one half of the Fourier transform is multiplied by *CTFP* and the other half by *CTFQ*, which is required to retain the Friedel relationship between the two halves of the transform. To treat the Fourier components on the other side of the particles, the reverse is done: i.e. *CTFQ* is applied to one half and *CTFP* to the other. Inside the computer, to reduce memory storage requirements, it is common practice to store only one half of the Fourier transform, with the other half being strictly determined by the Friedel relationship (with phases *α* and −α). Computationally, only *CTFP* is needed for the treatment of one of the pseudo-Friedel-related Fourier components and *CTFQ* for the other. Special care is needed to retain the Friedel relationship for Fourier components that lie exactly along the *x*^⋆^ or *y*^⋆^ axes. For example, *CTFP* should be applied only to $\left(+x^{★},0\right)$ and *CTFQ* to $\left(-x^{★},0\right)$. Practically this means replacing the conventional *CTFR* correction with two separate corrections, one using *CTFP* and the other using *CTFQ. CTFR* alters the amplitudes in the transform but leaves the phases unchanged, apart from a possible 180° flip. In contrast, *CTFP* and *CTFQ* alter the phases but leave the amplitudes unchanged. The transforms of the two respective single-sideband corrected images then sample the 3D transform in two positions that are slightly away from a central section due to Ewald sphere curvature, and these values must therefore be separately and correctly inserted into the 3D transform. This should provide a precise treatment of Ewald sphere curvature in any image over the bandwidth for which the spatial frequencies are delocalized by the defocus of the image. Since adjacent Fourier components are correlated in reciprocal space to an extent that is inversely related to particle size, there is one problem with our proposed single-sideband correction. At and near $x^{★}=0,$ there is a discontinuity when *CTFP* becomes *CTFQ*. The consequences of this discontinuity can be mitigated by rotating the *xy* coordinate system before application of *CTFP* and *CTFQ*, to obtain the corrected Fourier components in directions away from the discontinuity. In practice, since the discontinuity only exists near the axis separating *CTFP* from *CTFQ*, the calculation can simply be carried out in 4, 6 or 8 symmetric sectors and still be almost as efficient in computation as the initial 2 sector proposal but without the discontinuity. The final proposed procedure is presented schematically in Fig. 3. The deviation *Δz*^⋆^ from a flat central section due to Ewald sphere curvature (Fig. 1) can be calculated from trigonometry using a small-angle approximation

(5) $$\left(2/\lambda \right)\Delta z^{★}=1/d^{2}$$

so as an example, at 300 keV the 3 Å Fourier component (d=3 Å) is shifted by

$$\Delta z^{★}=\lambda /2d^{2}=0.02/18=1/900{\overset{˚}{\text{A}}}^{-1}$$

The separation along *z*^⋆^ between the two Fourier components recorded on opposite sides of the particle is then $2\Delta z^{★}=1/450\;{\overset{˚}{\text{A}}}^{-1}$. Single-sideband correction will thus make a big difference for particles bigger than 450 Å at resolutions beyond 3 Å using 300 keV electrons, and should also improve maps at lower resolutions. For 100 keV electrons (λ=0.037 Å), it would make the same improvement for particles half the size.

### 2.2 Single side-band application

**Fig. 2:**
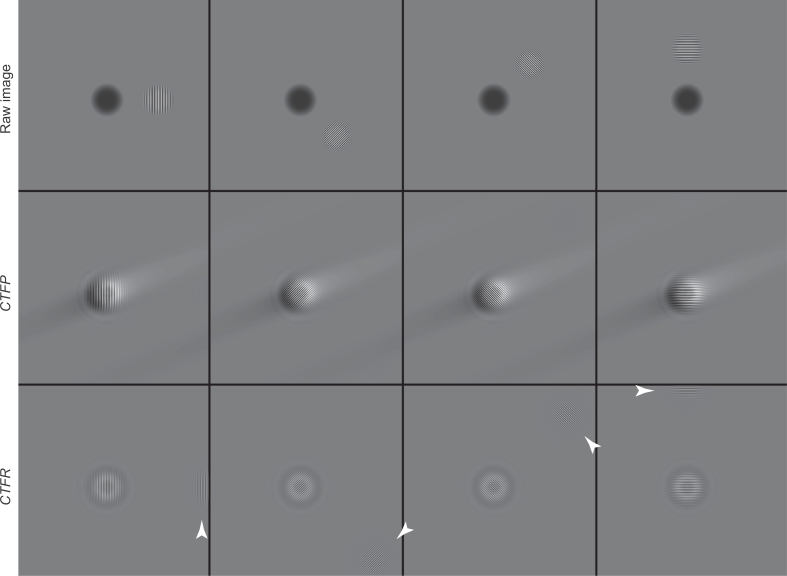
Simulation of a single-sideband image of an 80 Å particle at - 1.1 µm defocus with only one 3.8 Å Fourier component. The four top panels show four Fourier components in different directions relative to the *xy* axes, with a uniformly dark region to indicate the shadow of the particle at low frequency. The four middle panels show the result of applying the appropriate *CTFP*/*CTFQ* correction. The four bottom panels show the result of applying the conventional *CTFR*. The bottom panels also show how the fringes are moved in both the correct and incorrect directions, as indicated by the arrowheads. This figure shows a simplistic simulation to demonstrate the effect of the algorithm for single side-band correction. The application of *CTFP*/*CTFQ* was carried out using only one sector, rotated by 22° anticlockwise, so that all four 3.8 Å Fourier components would be treated correctly. This creates the diagonal low frequency halo seen in the four middle panels.

The theoretical procedure described above was applied to the simulated and real images shown in Figs. 2 and 4, using a program that multiplies the Fourier transform of an image by *CTFR* or *CTFP*/*CTFQ* in a single sector rotated by an arbitrary angle.

**Fig. 3:**
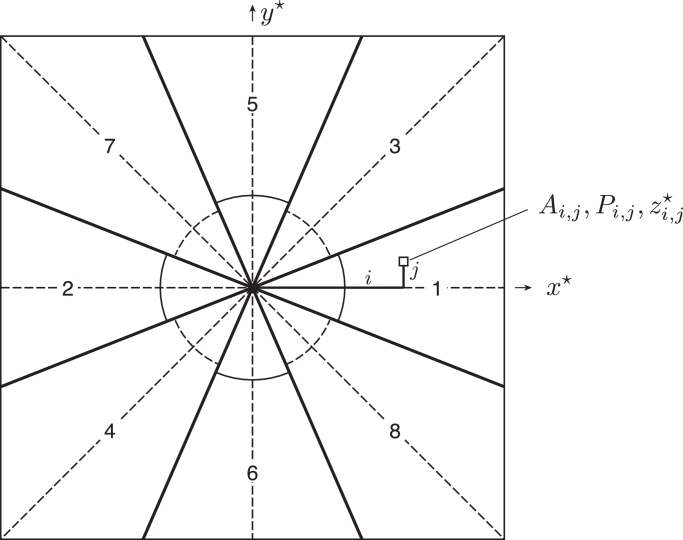
Proposed multi-sector procedure to allow precise extraction of the Fourier components so that they can be inserted into the 3D transform in their correct position, taking the Ewald sphere curvature into account.

**Fig. 4:**
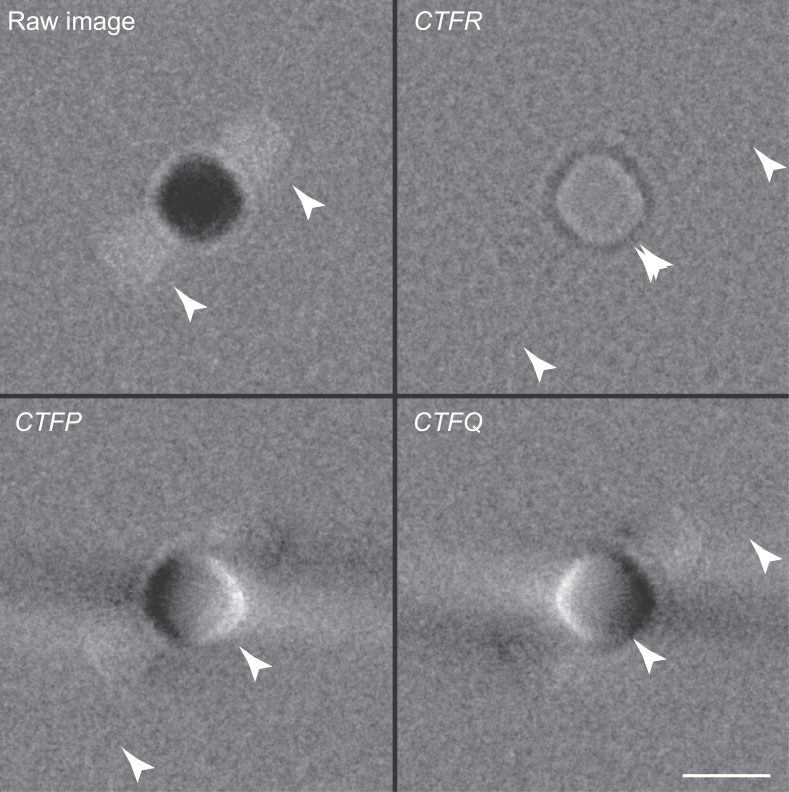
Application of procedure to an image of a gold nanoparticle in ice. Panels show the raw image of one gold particle with its two approximately Friedel-related diffraction spots, and the image after application of *CTFR, CTFP* and *CTFQ*. Application of *CTFP*/*CTFQ* was carried out using a single unrotated sector, which creates the low-resolution fringing seen in the bottom two panels. This image (no. 234638) was recorded with 300 keV electrons at 148600 ×  magnification, and - 1.105 µm defocus on a Falcon 3 detector in integrating movie mode. Arrowheads indicate the location of the 2.35 Å lattice fringes in each image and scale bar is 100 Å.The RMS amplitude at 2.35 Å in Fourier transforms of the regions marked by arrowheads was 257 and 255 in the raw image as well as in *CTFP* and *CTFQ*, whereas in *CTFR* the central RMS amplitude was 246 and the two ghost regions were 125 and 129 (in arbitrary units of intensity, all scaled the same).

## 3 Results

### 3.1 Single side-band CTF correction: Simulation

Fig. 2 shows a noise-free simulated image calculated using a defocus of 1.1 µm for a particle of diameter 80 Å that is oriented so that it diffracts only a single 3.8 Å Fourier component in one direction. It can be seen that the application of the proposed single-sideband *CTF* results in the movement of the Fourier fringe back to where it started out on the particle. Subsequent Fourier analysis of the masked image of the complex *CTF*-corrected particle allows the amplitude and phase of the single-sideband image to be extracted. By rotating the direction of application of *CTFP* and *CTFQ*, Fourier components in all directions, whether to the right or left, up or down, or at any arbitrary intermediate angle, can be treated correctly especially at low frequency. The 8-sector procedure proposed here is diagrammed in Fig. 3.

### 3.2 Single side-band CTF correction: Experimental demonstration

As a proof of principle, we wished to test the single side-band CTF correction method on experimental single-particle electron micrographs with strong, high-resolution spatial features that would be significantly affected by Ewald sphere curvature. Images of gold nanoparticles in ice, previously described in [5], are ideal for this purpose since they contain clearly delocalised side-bands from their crystalline reflection planes. We applied the single side-band correction algorithm to the defocused, high-resolution micrograph of gold nanoparticles whose diameter was 10 nm. The results are shown in Fig. 4, where the effect of the single side-band correction is compared with traditional *CTF* correction. The results show that for these imaging conditions (300 keV, −4 µm defocus) the signal strength at 2.35 Å is identical to that obtained using traditional *CTFR* correction except that individual sidebands are kept separate. We also note that the images in Figs. 2 and 4, when processed using traditional *CTFR* correction, show that half of the power goes back into the particle as desired and half is delocalised by twice what it was before. This point was emphasized by Downing & Glaeser [6] who noted that this effect is known as the twin image problem in reconstructing in-line holograms.

## 4 Discussion

It is well known that bright field phase contrast images of the weak phase objects that are typically obtained with biological structures in electron cryomicroscopy, can also be described as in-line Fresnel holograms [2]. They have typical Fresnel numbers around 50 depending on defocus, whereas defocussed diffraction patterns have Fresnel numbers much less than 1 [7]. In cryoEM, the contrast transfer function currently used to correct cryoEM images can be applied analytically, whereas typically the amplitudes and phases of the object are recovered from in-line holograms by an iterative numerical procedure [8] that is slow and computationally inefficient. Still, both of these established procedures result in a superposition of the two pseudo-Friedel-related Fourier components, which are thereby intermingled and can no longer be separately recovered. Here we have described an analytical procedure that is only slightly cumbersome computationally, but which is nevertheless mathematically rigorous. It can be equivalently thought of as a complex *CTF* correction, or as a resolution-dependent, single-sideband origin shift in the real space image, or as an analytical replacement for the iterative numerical procedure typically used in holographic phase retrieval. Its key feature is that it keeps the two pseudo-Friedel-related Fourier components separate, and avoids the superposition and subsequent ambiguity that occurs with conventional *CTF* correction or holographic phase retrieval. The conventional *CTF* approach not only puts the Fourier components into the 3D transform on a central section, and thus at the wrong *z*^⋆^ position, but it also adds them in with the wrong weighting. Beyond the resolution where the two pseudo-Friedel-related observations are not overlapped in the image, the weights should all be 1.0, yet conventional *CTF* processing puts some of them in with zero weight since the *CTF* has zeroes. This incorrect weighting is avoided using the approach described here. As a result, single sideband correction followed by appropriate masking of images recorded with adequate defocus, solves the so-called “Ewald sphere problem” in which two opposed Fourier components are superimposed in the image and therefore cannot be disentangled.

A formal proposal [9] for determination of the amplitudes and phases of the two pseudo-Friedel-related Fourier components involved deconvolution of the Fourier components obtained from two or more images of the same specimen at different defocus values. Radiation damage in the second and subsequent images makes this procedure less than ideal for biological specimens as the high-resolution features are progressively lost with increasing dose. A number of other papers have previously addressed the problem of “depth of focus” or “defocus gradient” between the top and bottom of the specimen. Jensen and Kornberg [10] suggested that the slices at different heights in the particle images could be calculated by using values for *CTFR* that are appropriate for each slice. This approach, later implemented for tomographic reconstruction as well [11], has a certain intuitive appeal but still introduces noise from the “out-of-focus” slices in a similar way to using the wrong *z*^⋆^ coordinate for insertion of the Fourier component into the 3D transform. Kazantsev et al. [12] also assume the images are projections, which is only an approximation. Wan et al. [13] restate the proposals of DeRosier [9] with slightly different mathematics. Wolf et al. [3] implemented two practical procedures to account for Ewald sphere curvature in Frealign. Their first procedure used a single side-band correction to insert the structure factor twice, each at the correct location in the 3D transform. Still, since no subsequent masking of the single sideband image was carried out, the wrong Fourier component is also inserted at each point. As more particles are added, the incorrect insertion will be localised on a tangential circle that surrounds the correct point of insertion and will eventually build up to create a systematic error in the 3D transform. In the second procedure, they performed an iterative, “reference-based” refinement, which performed less well than simple insertion when realistic noisy data was used in their simulation. Finally, Shang and Zhou [14] reviewed the above proposals including a discussion of dynamical scattering using the simplified model of Yanga et al. [15]. While these papers all touch on the problem, none of them experimentally separate the two pseudo-Friedel-related components clearly so that they can each be inserted into the 3D transform at the correct *z*^⋆^ position with the correct weight. The algorithm proposed here does this.

The defocus necessary to record the two pseudo-Friedel-related Fourier components separately in the image is *Dd*/2*λ*, where *D* is the particle diameter, *d* is resolution and *λ* is the electron wavelength. So, for a 100 Å particle, a defocus of at least 1 µm in magnitude is required to separate physically the Fourier components beyond 4 Å resolution. For larger particles, such as a 1000 Å virus, larger (10 µm) defocus is needed. Fig. 6 shows the relationship between particle size, defocus and electron energy graphically. This requirement for a finite amount of defocus is not incompatible with use of a Zernike phase plate, for which zero defocus and zero *C_s_* would be optimal. A phase contrast image with *π*/2 phase shift from a Zernike phase plate and defocus of 1–2 µm would still separate the two high resolution Fourier components, yet retain a strong low-resolution envelope function out to the first transfer function zero at 20–30 Å resolution.

Ultimately, the accuracy of single side-band correction is limited by the accuracy with which the transfer function fitting parameters are determined, just as it is for traditional *CTF* correction. Fortunately, the values obtained for defocus and astigmatism using established programs such as CTFFIND4 [16] or GCTF [17] are the same parameters needed for single sideband correction. The defocus given by CTFFIND4 or GCTF is what defines the particle origin in *z* for the single sideband phase correction just as it does in conventional *CTFR* correction, and as long as this is accurate to within 50–100 Å, the resolution will not be affected. In practice, the origin will be approximately at the centre of mass of the particle, even if the particle is 1000 Å in diameter, since the *CTF* minima fitted in CTFFIND4 arise from all the atoms.

**Fig. 5:**
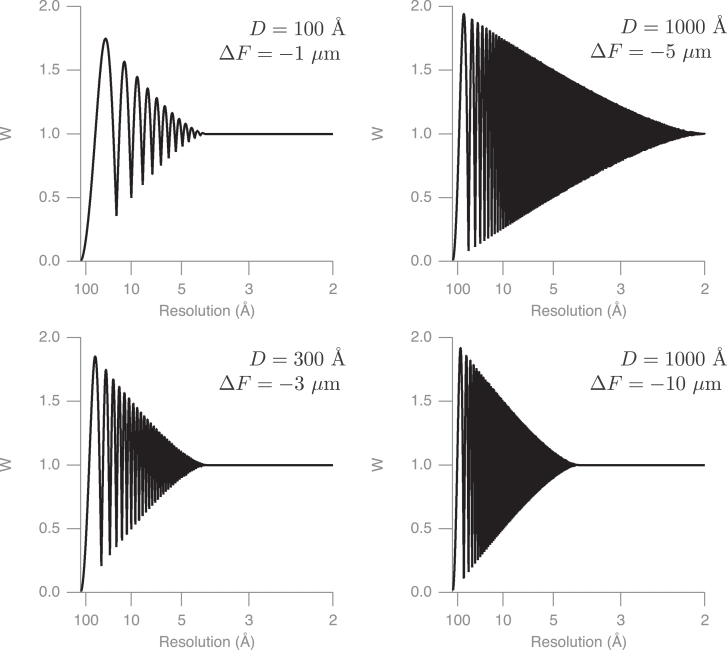
Plot of the weighting function W that takes into account the overlap of the two pseudo-Friedel-related components at lower resolution. There is no overlap for Fourier spacings where *ΔF* > *Dd*/2*λ*, so W=1.0 beyond that. At very low resolution, where the two components are largely overlapped, the value of *W* is equivalent to that of a conventional *CTF*. The defocus *ΔF* and the particle size *D* control the bandwidth of the transition at a given energy, here 300 keV (λ=0.02 Å).

This paper has outlined a theoretical procedure and demonstrated the method with a proof of principle experiment. In practice, the sequence of computations required for 3D structure determination using single-sideband correction might follow the following steps:

1. Extract boxed particles from raw images using a box size large enough to include Fourier components to the maximum anticipated resolution.
2. Calculate the Fourier transform of the full area within each box.
3. Multiply the Fourier transform by *CTFP*/*CTFQ* in sectors using the proposed procedure.
4. Inverse-transform each of the resulting modified transforms.
5. Apply a soft-edged box to mask out the particle and maximise the signal-to-noise ratio.
6. Fourier transform the masked areas.
7. Combine the appropriate parts of the resulting Fourier transforms from each of the sectors after steps 5 and 6, to give a two-dimensional transform in which each Fourier component is described by *AMP*(*i, j*), *PHS*(*i, j*) and *ZSTAR*(*i, j*) for values of *i* and *j* that extend from negative to positive values, where *AMP, PHS* and *ZSTAR* represent the discrete valued amplitude, phase and distance from the central section.
8. Insert the individual Fourier components into the 3D transform at the correct position in reciprocal space, with a weighting that takes into account the overlap of the two pseudo-Friedel-related components at low resolution. This is given by the following equation (see also Fig. 5 which plots *W* for several examples):
  (6) W=1+A(2|sinχ|−1)
  where *A* is defined as the degree of overlap between the two pseudo-Friedel-related sidebands, which varies from 0.0 to 1.0 according to
  (7) $$A=\left\{ \begin{array}{c} \frac{2}{\pi }\left[\arccos\frac{2\Delta F\lambda }{dD}-\frac{2\Delta F\lambda }{dD}\sin\left(\arccos\frac{2\Delta F\lambda }{dD}\right)\right],\\ \;\;\text{for}\;0<\Delta F<Dd/2\lambda \\ 0,\;\text{for}\;\Delta F>Dd/2\lambda \;\text{(no}\;\text{overlap)} \end{array}\right.$$

**Fig. 6:**
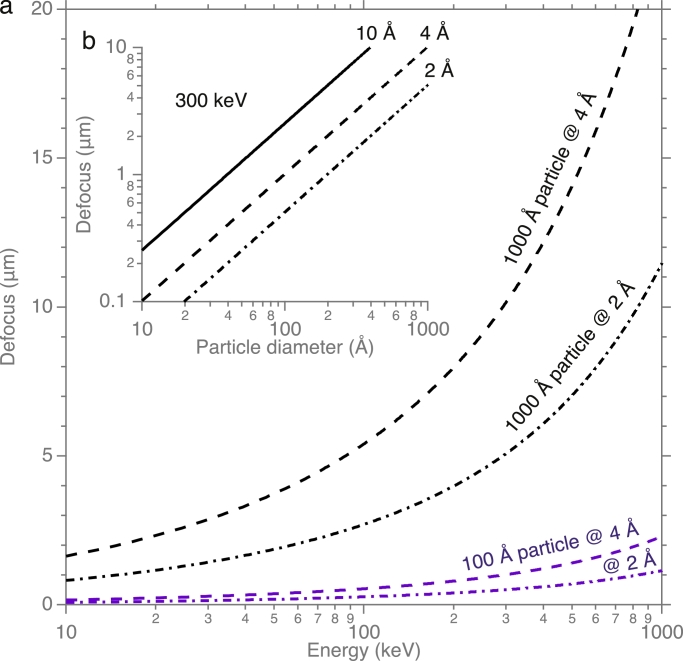
Plots of the relationship between particle size, defocus and electron energy. Plots show the defocus required (*ΔF* > *Dd*/2*λ*) to ensure that all Fourier components from a particle of diameter *D*, beyond the indicated resolution, *d*, are completely separated in the image. Panel (a) plots this relationship vs electron energy and (b) shows the dependence on the size of the particle.
9. The weighted summation used to calculate the structure factor at each point in the 3D transform in conventional treatment of the *CTF* is given by
  (8) $$A_{i,j,k}=\frac{\sum_{n=1}^{N}CTF_{n,i,j}A_{n,i,j,k}}{\sum_{n=1}^{N}CTF_{n,i,j}^{2}+w}$$
  where *N* is the number of particles and *w* is a Wiener factor. The corresponding summation in the 3D transform in the single sideband case is
  (9) $$A_{i,j,k}=\frac{\sum_{n=1}^{N}\sum_{p,q}A_{n,i,j,k}}{\sum_{n=1}^{N}\sum_{p,q}W_{n,i,j}^{2}+w}$$
  where *W*_*n, i, j*_ is the value given by (6) for particle *n*, and *p, q* are the two components from opposite sides of the Ewald sphere. Note that no weighting term is necessary in the numerator of (9) because the components *p* and *q* implicitly interfere constructively or destructively where the two terms overlap to create the correct weight, but the explicit weighting term is still required in the denominator.
10. In practice, it will be useful to calculate two sets of 3D maps in which the Ewald sphere correction Δz* is applied in the correct and incorrect directions, so that the success of the procedure can be measured. For example, the resolution (as assessed e.g. by Fourier shell correlation) of the map with the components inserted correctly should improve relative to a map with a flat Ewald sphere (conventional *CTFR*) and deteriorate when inserted incorrectly. Having two maps with insertion at positive and negative Δz* would also allow direct validation of the absolute hand of the structure since the map calculated with the correct direction of curvature will correlate better with a correctly handed model of the structure.

Another advantage of the use of this single side-band *CTF* correction is that the Ewald sphere curvature or “depth-of-field” problem that normally gets worse at lower accelerating voltages [18], [19], is no longer a limitation, so the way is now open for development and use of longer wavelength, lower energy electrons. This might become an advantage since electron scattering theory suggests that the ratio of elastic to inelastic scattering increases by about 30% for 100 keV electrons in comparison with 300 keV electrons [20] so more structural information is potentially available per unit damage. Other costly technical limitations of making high-energy electron microscopes, such as ultrastable, high-voltage and high current power supplies, and lead shielding to absorb X-ray emission from the column optics, also point to the development of low-energy microscopes for cryoEM. The development of electron lens aberration correction [21], which allows atomic resolution at low energy, and the recent development of purpose-built highly corrected low-energy electron microscopes (e.g. SALVE 3 project) mean that the electron optics should not prevent atomic resolution imaging of biological specimens at lower electron energy [22]. While multiple scattering and the total electron scattering cross section will still put an ultimate limit on the thickness of a specimen that can be imaged at high resolution (the mean free path in ice for 100 keV electrons is  ≃ 1500 Å [23], and roughly twice the value at 300 keV), even this can be partially compensated by the judicious use of chromatic aberration correction to bring the inelastic-elastic scattering events back in focus [24]. In addition, since the Ewald sphere is curved in a known direction in the third dimension (towards the source), and unlike in X-ray crystallography the phases as well as amplitudes have been measured, the procedure we describe also automatically determines the absolute hand of the specimen without the necessity of recording tilt pairs, which is required for structures determined from images processed with conventional *CTFR* correction.

## 5 Conclusions

The work described here proposes an improved approach to data collection and image processing in high-resolution biological electron cryomicroscopy. We have demonstrated a method which allows the observed Fourier components to be inserted in the 3D transform at the correct position and with the correct weight. Surprisingly, all current structures determined by cryoEM have, to some extent, inserted the Fourier components incorrectly, particularly at high resolution. This means that the processing of previous datasets may be improved using this algorithm, and future data collection strategies, including those using phase plates, will benefit. This method will thus help to expand the range of specimens amenable to high resolution imaging, allow the use of electrons with lower energy, and potentially improve the resolution of any structure determined by cryoEM.
